# Serum Protein Electrophoretic Pattern in Neonatal Calves Treated with Clinoptilolite

**DOI:** 10.3390/molecules23061278

**Published:** 2018-05-26

**Authors:** Simona Marc, Danijela Kirovski, Călin Mircu, Ioan Hutu, Gabriel Otavă, Cristina Paul, Oana Maria Boldura, Camelia Tulcan

**Affiliations:** 1 Faculty of Veterinary Medicine, University of Agricultural Sciences and Veterinary Medicine “King Michael I” of Banat Timişoara, Calea Aradului 119, 300645 Timişoara, Romania; simo20_med@yahoo.com (S.M.); calinmircu@yahoo.com (C.M.); ioan.hutu@yahoo.com (I.H.), gabiotava@yahoo.com (G.O.), oanaboldura@gmail.com (O.M.B.); 2 Faculty of Veterinary Medicine, University of Belgrade, Bulevar Oslobodenja 18, 11000 Belgrade, Serbia; dani@vet.bg.ac.rs; 3 Faculty of Industrial Chemistry and Environmental, University Politehnica Timisoara, Carol Telbisz 6, 300001 Timisoara, Romania; cristina.paul@upt.ro

**Keywords:** electrophoresis, calves, clinoptilolite, γ-globulin

## Abstract

The objective of our study was to determine the effects of clinoptilolite supplemented in colostrum on the blood serum protein electrophoretic pattern of new-born calves. Methods: Romanian Black and White new-born calves involved in the study were divided into 3 groups: the control group (C) that received colostrum without clinoptilolite, and experimental groups I (E1) and II (E2) that received colostrum supplemented with 0.5% and 2% clinoptilolite, respectively. The concentration of total protein and protein fractions (albumin, α1-globulin, α2-globulin, β-globulin and γ-globulin) were analyzed by electrophoresis on cellulose acetate. Results: At hour 30 after birth, concentrations of γ-globulins, β-globulin and total protein in E1 group of calves were higher than in control group by 42.11% (*p* < 0.05), 28.48% (*p* > 0.05) and 18.52% (*p* > 0.05), respectively, and were higher, but not significantly, in group E2 compared to the control group. This was in accordance with a significant lower albumin/globulin ratio in groups E1 and E2 (29.35%, *p* < 0.05 and 35.87%, *p* < 0.05, respectively) than in control group at 30 h postpartum, which indicates an obvious increase of the globulins fraction in experimental groups. The conclusion: Clinoptilolite was effective in improving passive transfer in new-born calves, but it was more effective if added in colostrum with a dose of 0.5% than with a dose of 2%.

## 1. Introduction

Gut immunoglobulins absorption is time limited, since the permeability of enterocytes is highest at birth, and is reduced by 50% during the next 6 h, due to intestinal cells maturation in newborns, i.e., mature cells population does not alow macromolecules transfer and their lysozyme activity increases progressively leading to enzymatic digestion of immunoglobulins [1,2].

Factors that affect the level of colostrum immunoglobulins absorbtion are time of the first colostrum feeding after birth, quality and quantity of colostrum, pattern of taking the colostrum, bacterial contamination of colostrum, dam’s lactation number, breed, season of calving, dam’s dry period length, maternity microclimate and immunity status of dam. If one or several of those factors are disturbed, the failure of the transfer of passive immunity (FPT) in newborns occurs. The incidence of FPT is high in young ruminants leading to a high incidence of neonatal morbidity and mortality, with possible reduced weight gain and increased drug use which cause huge economic loss on dairy farms [3,4,5,6].

Colostrum quality improvement may enhance immunoglobulin absorption [3]. Supplementation of colostrum with clinoptilolite, a natural zeolite, is a natural method to improve immunoglobulin absorption in newborn calves [7,8,9,10]. Zeolites are crystalline, hydrated aluminosilicate of alkali and alkaline earth cations having three dimensional structure [11]. Due to their unique properties (cation exchange, adsorption or acting as molecular sieves, catalytic, dehydration/rehydration and biological reactivity), zeolites are used in a wide range of biological processes in human and veterinary fields where they have antiviral effects [12], antitumoral effects [13,14] and antioxidant effects [15]. In addition, they are used in the prevention and/or treatment of certain farm animal diseases [16,17].

Immunoglobulin concentrations in blood serum can be evaluated through electrophoretic techniques, due to the migration of the last fraction, γ-globulins. Serum electrophoretic method is not commonly used in bovine practices, although in small animals it is used as a laboratory diagnostic technique for protein metabolism. Studies in ruminants revealed factors that may have influenced the results, such as the physiological status of the animal and its age, as well as laboratory pre-analytical preparations of the samples [18,19,20,21].

The aim of this study is to analyse the effects of short-term clinoptilolite supplementation added in two different doses in colostrum on serum protein levels in newborn calves, especially on the immunoglobulins, analysed by electrophoretic method.

## 2. Results

**Figure 1:**
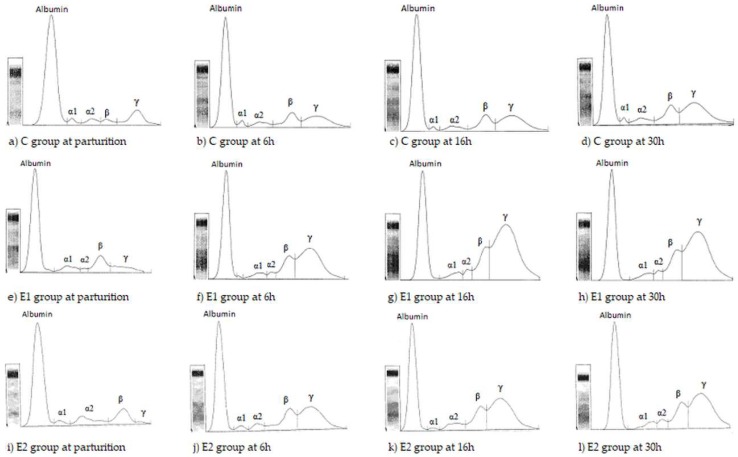
Representative serum protein electrophoretograms observed in calves from C, E1 and E2 groups during parturition, at 6, 16 and 30 h postpartum. (**a**) C group at parturition. (**b**) C group at 6h postpartum. (**c**) C group at 16h postpartum. (**d**) C group at 30h postpartum. (**e**) E1 group at parturition. (**f**) E1 group at 6h postpartum. (**g**) E1 group at 16h postpartum. (**h**) E1 group at 30h postpartum. (**i)** E2 group at parturition. (**j**) E2 group at 6h postpartum. (**k**) E2 group at 16h postpartum. (**l**) E2 group at 30h postpartum.

The calves in all groups had a significant increase in the concentration of total protein in time, but there was no significant difference between groups 30 h postpartum (*p* > 0.05). Albumin concentrations did not significantly change after consuming colostrum, and were no different between groups at 30 h postpartum (*p* > 0.05). The time and group influence on α1 and α2-globulin fractions was not recorded at 30 h postpartum, except for the α1 globulin fraction in E2 that decreased at 30 h as compared to 6 h (*p* < 0.02). β-globulin fractions showed significant increases in all groups (E1, E2, C) in comparison with birth time (*p* < 0.05), but there were no statistical differences in concentration of this fraction between groups 30 h postpartum (*p* > 0.05). γ-globulins percentage was significantly higher in E1 compared to the control group at 30 h of life (*p* < 0.05), but not in the E2 group (*p* > 0.05) (see Table 1 and Figure 1).

**Figure 2:**
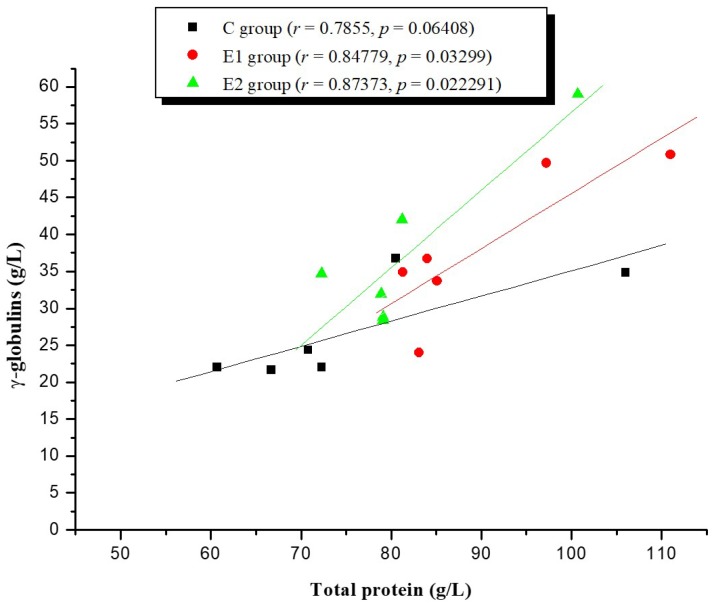
Relation between total proteins and γ-globulins concentrations in calves at 30 h postpartum.

In addition, there was a positive correlation between total protein and γ-globulin concentrations at 30 h postpartum in groups E1 and E2, but not in group C (Figure 2).

## 3. Discussion

Serum total protein concentration is an indicator of the successful transfer of passive immunity in calves, because there is a positive relationship between such a parameter and immunoglobulins. The adequate passive transfer of immunoglobulins in calves is considered when total protein concentrations at 24 h postpartum is more than 52 g/L, which is equivalent to 10 g/L IgG [6]. Thus, there was no FPT in any of examined groups of calves in our study, since all of calves had a concentration of total protein higher than 54 g/L and serum γ-globulins higher than 10 g/L starting with the administration of first colostrum.

The colostrum used in this experiment was from Romanian Black and White cows that were in 2nd to 4th lactation. Weaver et al. (2000) [6] suggested that cows that were in 1st to 4th lactation provide high-quality colostrum to their calves, meaning that the calves in our study received high-quality colostrum. According to our previous results based on chemical composition of colostrum of cows [22], compared to high-yielding breeds (Holstein), autohtonious breeds, even if they have lower daily milk production (12 L in our study), have a better quality of colostrum. Total protein levels determined in blood serum of calves reflect both the level of colostrum ingested and the effects of clinoptilolite on immunoglobulin absorption. The method of colostrum administration can affect the efficiency of colostral immunoglobulins transfers, since the highest percent of calves that suffer from FPT are those that suckle their dams (61.4%), while only 19.3% of calves with FPT are fed by bottle and 10.8% are fed by tube [4]. The calves from our study received their first colostrum within 2 h after parturition, because immediately after birth calves are not able to ingest adequate amounts of colostrum and because they need some time to acclimate to an extra uterine environment. According to the literature [6], the time of ingestion is positively correlated with the cessation of colostral immunoglobulins absorption from the intestine. 

After consuming colostrum, serum γ-globulins fractions increased significantly in all groups of calves, in comparison with time of birth, when the serum levels of γ-globulins were almost nonexistent (*p* < 0.05), which highlights the lack of transplacental transfer of immunoglobulins and the importance of colostrum on passive transfer success. In addition, at 30 h after birth, experimental groups had higher concentration of serum γ-globulins fractions compared to control, significant in group E1 (*p* < 0.05).

Data from the literature offers us some possible explanations of clinoptilolite action on intestinal absorption of colostral immunoglobulins: (a) reduced negative impact of food decomposition process in intestines on mucosal epithelial cells that are involved in absorption of intact molecules of colostrum immunoglobulins; (b) possible adsorption of enteropathogens that colonize the digestive tract of the calf in the first hours after parturition thus limiting its attachement to the intestinal cell-membrane receptors and (c) increased the amount of absorption by increasing the length of enterocytes microvilli and reducing diameter thereof [16,23]. 

Decreased albumin level in experimental groups compared with control group showed that the A/G is in favor of globulins, which means a high level of absorption of colostral γ-globulins during the first 30 h postpartum. 

A/G is another parameter that highlights changes in serum globulin concentrations in calves. Thus a fall in the A/G in the first hours after birth in calf serum signifies an increase in the globulin fraction. The significant decrease in the A/G at 30 h postpartum recorded in E1 and E2 groups indicates a better passive transfer of γ-globulins in calves from groups E1 and E2, possibly due to clinoptilolite added in colostrum. 

Analyzing all five protein fractions, only the last one, γ-globulin, had a proportional increase time/dose, while the others increased inconstant. The α1 and α2 globulin fractions recorded variations after the administration of colostrum to all studied calves without influence of clinoptilolite. Higher β-globulin fractions in the experimental groups (E1, E2) as compared to control group (E1/C: +28.48%, E2/C: +18.52%) (*p* > 0.05) at 30 h after birth, means that the administration of clinoptilolite resulted in a significant increase in immunoglobulins A and M, because such types immunoglobulins have β globulinic mobility [2,24]. Our results were similar those of Reference [7,8] and different from those of Reference [5]. Mohri et al. (2008) suggested that following clinoptilolite supplementation at a rate of 2% for 48 h in colostrum and 1% for 14 days in colostrum and milk, serum total protein, γ and β globulin, except albumin were not significantly affected by supplementation. A possible explanation of increased albumin might be found in ensuring a more efficient digestion of milk proteins and a higher uptake of amino acids, which increased the hepatic albumin synthesis [5].

The interval chosen for blood serum collection (0, 6, 16 and 30 h postpartum) represents the interval of 4–6 h after administration of colostrum with/without clinoptilolite, which is the necessary time for the absorption of macromolecules in the intestine. We have stopped examination at 30 h postpartum (that means 6 h after administration of colostrum that occured 24 h postpartum), because it was reported that until hour 36 postpartum, the calf’s small intestine enterocytes would nonselectively absorb macromolecules [6]. We know from data from the literature that after 48 h postpartum, the values of calves gammaglobulins tend to stabilise and at about 4 weeks of age calves starts their own immunoglobulin synthesis [2,7,8,9,25,26]. Administration of clinoptilolite for a longer period of time (14 days) did not influence the level of calf’s serum γ-globulins [5,27].

There are many factors that influence serum immunoglobulins concentration in calves during the first 24–48 h postpartum. One is the apparent efficiency of absorption (AEA), the efficiency with which ingested colostral IgG is absorbed in circulation. Absorption efficiency is apparent because about 50% of IgG absorbed will pass in extravascular spaces, therefore, theoretically, the AEA goes up to 50% [28]. The absorption of IgG intake is effective when the AEA mean is around 25–35% [28]. The AEA observed in this study was effective for all groups, but slightly better for calves in group E1.

Although the concentration of γ-globulins fraction in blood serum of control group (26.95 ± 2.84 g/L) was high enough to protect the newborn calves from infection, the calves in this group had more gastraintestinal disorders (diarrhoea) in the first 28 postnatal days and the average weight was lower than in calves from experimental groups at 45 days postpartum [29]. Such data and those of [27,30] highlights the idea that health status of calves in their first weeks of life is influenced by the level of serum γ-globulins. 

Some mechanisms supporting the effect of the dietary use of zeolites on diarrhoea syndrome include: (a) possible stimulation of the defense line of the intestinal tract, causing the animal to produce more antibodies; (b) acting as a water adsorber or having a retarding effect on the intestinal rate [31]; (c) regulation of gut pH [11]; (d) binding of enterotoxins implicated in gastrointestinal disturbances; (e) changing metabolic acidosis through effects on osmotic pressure in the intestinal lumen [16]; (f) having adsorption effects on bile acids, one of endogenic causes of diarrhoea, as well as on aflatoxin B_1_ and glucose, that in high content in intestinal fluid acts as irritant factor and whose transport through the intestinal cells is reversed during diarrhoea [32]; (g) possible adsorption of dietary substances, which may result in intestinal hypersensitivity to feed antigens, possible maintaining or restoring the digestive enzyme activity [16,33]. 

The beneficial impacts of clinoptilolite on protein metabolism were demonstrated by the determination of hormones which act by stimulating the protein synthesis (insulin) or by increasing protein catabolism (thyroid hormones). Adding of natural zeolite (clinoptilolite) to colostrum caused considerable increase of IGF-1 and insulin in the blood serum of newborn piglets [34] and decreased thyroid hormone concentrations in newborn calves [35]. Such results indicate the possible beneficial effect on timely regulation of energy balance in newborns, by increasing the rate of resorption of nutrients in colostrum, providing newborn animals with sufficient energy. 

It is well known that adding mineral adsorbant into feed has beneficial effects. Mumpton (1999) described the role of zeolites in the gastrointestinal tract where they actas an ammonium reservoir, thereby allowing the animal to use ingested nitrogen more efficiently. Also ammonium containing zeolite may support the growth of nitrogen-loving bacteria that contribute to the health of the animals. This property of zeolites is important in young animals because their feed contains a high level of proteins and it may be concluded that zeolite could be vital for the digestion process and nutritive substances absorption even after the colostrum period [11].

## 4. Materials and Methods

### 4.1. Animals Experiment

The study was carried out on 18 newborn Romanian Black and White calves in Didactic Farm of Banat’s University of Agricultural Sciences and Veterinary Medicine “King Michael I” in Timişoara. The study was performed in compliance with national (471/2002) and international (Directive 86/609 CEE) laws regarding animal welfare and ethics in animal experiments. The approval of experiments was given by University Coomision of Ethics , USAMVBT-PG-001-R021/2014. Calves were separated from the dams within 20–30 min after parturition, and were weighed and transferred to the calf area. Calves were divided into 3 groups based on birth order: Control group (C, *n* = 6) and two experimental groups (E1, *n* = 6; E2, *n* = 6). Calves were bottle fed with the first colostrum (1.5 L) within 2 h after birth and after that two times daily, at 12 h interval, with the same quantity of colostrum that was milked at 12 h interval. We have chosen bottle feeding, which is the most common feeding on commercial dairy farms, due to the fact that in that way we could control the quantity of colostrum ingested by the calf and we could add clinoptilolite to colostrum.

Calves in first experimental group (E1) received colostrum with 0.5% clinoptilolite, within 2 h after birth, at 12 and 24 h. In the second experimental group (E2), calves received 2% clinoptilolite in the same interval as the first experimental group. 

The blood samples were collected from jugular vein in vacutainer tubes prior to colostrum intake, at 6, 16 and 30 h after birth. Blood serum was obtained after centrifugation of the samples at 3000× *g* for 5 min and stored at −20 °C until analysis.

### 4.2. Clinoptilolite

The commercial product of clinoptilolite (Min-a-Zel S, Patent Komerc, Belgrade, Serbia) was used in present study. The Min-a-Zel S, as a natural zeolite, contains minimum 85% clinoptilolite. Granulometry of clinoptilolite is 100% <300 µm. The chemical analysis of the product was determined at ITNMS (Institute for the Application of Nuclear Energy), Belgrade, Serbia.

### 4.3. Quantitative Analysis of Total Protein

Serum total protein content was determined on clinical chemistry automatic analyzer (EOS BRAVO FORTE Hospitex Diagnostics, Fiorentiono, Italy) by biuret colorimetric method using dedicated commercially reagent kit, REF. 4001950L (Hospitex Diagnostics, Fiorentino, Italy). Internal quality control was performed with reference materials (calibrator, control serum with normal and pathological values) from the same producer (REF 40011935, 40011925 respectively 40011930). Colostrum IgG were analyzed by radial immunodifussion method by commercially available kits (INEP ZEMUN, Belgrade, Serbia).

### 4.4. Electrophoresis Analysis of Serum Protein Fractions

Protein fractions (albumin, α1-globulin, α2-globulin, β-globulin and γ-globulin) were analyzed by electrophoresis on Mylar^®^ 35 × 76 mm backed cellulose acetate strips by kits for determining serum protein (code SRE174K, INTERLAB, Rome, Italy) with Genio electrophoresis automatic analyzer. 30 μL of the sample was subjected to the microtechnique assay with following method parameters: sample application time 10 s, migration time 15 min., staining time 300 s, destaining time 180 s, clearing time 120 s, drying time 620 s, migration voltage 140 V with an optical density scanning green light.

For controlling measurement accuracywe used normal serum control (REF:SCE123A) and pathological serum control (REF:SCE126A). The automated system was regularly monitored for accuracy and precision in accordance with ”Good laboratory practice” guidelines.

Serum concentration of protein fraction (g/L) was obtained according to relationship:

$$\mathrm{Concentration}\;\mathrm{of}\;\mathrm{protein}\;\mathrm{fraction}\;\left(g/L\right)=\frac{\mathrm{total}\;\mathrm{protein}\;x\;\mathrm{protein}\;\mathrm{fraction}}{100}$$

Albumin/globulins ratio (A/G) was obtained according to relationship:

$$A/G\;\mathrm{ratio}=\frac{\mathrm{albumin}\;\mathrm{concentration}}{\sum^{\text{​}}\mathrm{globulins}\;\mathrm{concentration}}$$

Apparent efficiency of absorption (AEA) of γ globulins was calculated by taking into account the quantity and quality of colostrum ingested and the volume of calf serum. Plasmatic volume was calculated as being 9.1% of body weight [22].

$$\mathrm{AEA}\;\left(\text{\%}\right)=\frac{\mathrm{serum}\;\mathrm{IgG}\;\mathrm{concentration}\;x\;\mathrm{plasmatic}\;\mathrm{volume}}{\mathrm{IgG}\;\mathrm{consumed}}\;\times \;100$$

### 4.5. Statistical Analysis

The results obtained were processed using the statistical software MINITAB 13 (licenced for USAMVBT, Timisoara, Romania). Values are expressed as means with standard error (x ± SE). Results were evaluated using a one way analysis of variance (ANOVA) with 3 variables (calf, treatment, and time). Whenever the ANOVA revealed significat *F* values for such variables, a *t*-test was used for pos hoc comparation. Results were deemed as statistically significant when a *p* value was <0.05.

## 5. Conclusions

Supplementation of colostrum with clinoptilolite has a positive influence on serum γ-globulin fractions and total protein concentration in neonatal dairy calves, with better results obtained by adding 0.5% rather than 2% clinoptilolite.

## Figures and Tables

**Table 1:** Total protein concentration and protein fractions (x ± SE; g/L) in blood serum of calves.

Parameters	Group	Parturition	6 h	16 h	30 h
Total protein (g/L)	C	42.65 ± 6.26	55.40 ± 5.28	74.03 ± 3.89 *^,^**	76.17 ± 6.54 *^,^**
	E1	46.40 ± 1.32	72.23 ± 7.12 *	84.35 ± 4.50 *^,^**	90.28 ± 4.75 *^,^**
	E2	38.28 ± 5.65	54.73 ± 10.73	66.40 ± 12.53 *	81.88 ± 3.96 *^,^**
Albumin (g/L)	C	32.86 ± 4.67	27.69 ± 3.74	32.47 ± 3.28	36.43 ± 3.57
	E1	33.36 ± 1.32	34.72 ± 8.52	32.88 ± 4.33	34.86 ± 1.97
	E2	28.69 ± 4.68	25.94 ± 5.02	23.42 ± 3.83	29.16 ± 3.15
α1-globulin (g/L)	C	1.40 ± 0.21	1.54 ± 0.33	2.42 ± 0.40	2.71 ± 0.48
	E1	2.54 ± 0.61	2.06 ± 0.84	4.23 ± 1.78	4.04 ± 0.56
	E2	0.94 ± 0.18	1.12 ± 0.28	0.83 ± 0.19	2.32 ± 0.60 *
α2-globulin (g/L)	C	2.43 ± 0.57	3.82 ± 1.31	4.23 ± 1.69	2.24 ± 0.36
	E1	4.15 ± 2.01	8.10 ± 1.85	4.48 ± 0.57	2.98 ± 0.19
	E2	4.01 ± 0.32	5.72 ± 1.12	5.33 ± 1.67	3.66 ± 0.96
β-globulin (g/L)	C	4.41 ± 1.34	6.89 ± 1.58	7.03 ± 1.17	7.83 ± 0.85 *
	E1	5.13 ± 1.20	8.56 ± 1.27	8.71 ± 0.40	10.06 ± 0.74 *
	E2	4.08 ± 0.83	8.14 ± 1.24 *	8.71 ± 2.49	9.28 ± 0.52 *
γ-globulin (g/L)	C	1.50 ± 0.48	15.86 ± 4.06 *	27.83 ± 3.85 *^,^**	26.95 ± 2.84 *^,^**
	E1	0.68 ± 0.26	18.68 ± 3.56 *	34.03 ± 3.32 *^,^**	38.30 ± 4.19 *^,^**^,a^
	E2	0.57 ± 0.18	13.81 ± 4.87 *	28.01 ± 7.48 *^,^**	37.47 ± 4.77 *^,^**
A/G	C	3.90 ± 0.67	1.16 ± 0.24 *	0.85 ± 0.14 *^,^**	0.92 ± 0.06 *^,^**
	E1	3.04 ± 0.50	1.02 ± 0.30 *	0.66 ± 0.12 *^,^**	0.65 ± 0.08 *^,^**^,a^
	E2	2.88 ± 0.27	0.92 ± 0.08 *	0.60 ± 0.06 *^,^**	0.59 ± 0.10 *^,^**^,a^

Significant differences (*p* < 0.05) inside the group: * vs. parturition, ** vs. 6 h, Significant differences (*p* < 0.05) between the groups: ^a^ at 30 h (E1 vs. C).
